# Prognostic Analysis of Patients with Ebola Virus Disease

**DOI:** 10.1371/journal.pntd.0004113

**Published:** 2015-09-23

**Authors:** Xin Zhang, Yihui Rong, Lijian Sun, Liming Liu, Haibin Su, Jian Zhang, Guangju Teng, Ning Du, Haoyang Chen, Yuan Fang, Wei Zhan, Alex B. J. Kanu, Sheku M. Koroma, Bo Jin, Zhe Xu, Haihan Song

**Affiliations:** 1 PLA General Hospital, Beijing, China; 2 Beijing 302 Hospital, Beijing, China; 3 BGC Biotechnology Research Center, Jinan, Shandong, China; 4 Jui Government Hospital, Freetown, Sierra Leone; 5 Emergency Center, Shanghai East Hospital, Tongji University School of Medicine, Shanghai, China; Armed Forces Health Surveillance Center, UNITED STATES

## Abstract

**Background:**

The Ebola virus causes an acute, serious illness which is often fatal if untreated. However, factors affecting the survival of the disease remain unclear. Here, we investigated the prognostic factors of Ebola virus disease (EVD) through various statistical models.

**Methodology/Principal Findings:**

Sixty three laboratory-confirmed EVD patients with relatively complete clinical profiles were included in the study. All the patients were recruited at Jui Government Hospital, Sierra Leone between October 1st, 2014 and January 18th, 2015. We first investigated whether a single clinical presentation would be correlated with the survival of EVD. Log-rank test demonstrated that patients with viral load higher than 10^6^ copies/ml presented significantly shorter survival time than those whose viral load were lower than 10^6^ copies/ml (P = 0.005). Also, using Pearson chi-square test, we identified that chest pain, coma, and viral load (>10^6^ copies/ml) were significantly associated with poor survival of EVD patients. Furthermore, we evaluated the effect of multiple variables on the survival of EVD by Cox proportional hazards model. Interestingly, results revealed that patient’s age, symptom of confusion, and viral load were the significantly associated with the survival of EVD cases (P = 0.017, P = 0.002, and P = 0.027, respectively).

**Conclusions/Significance:**

These results suggest that age, chest pain, coma, confusion and viral load are associated with the prognosis of EVD, in which viral load could be one of the most important factors for the survival of the disease.

## Author Summary

The current outbreak of Ebola virus disease (EVD) in West Africa is the largest and most complex Ebola outbreak since the virus was first discovered in 1976. Factors affecting the survival of the disease remain unclear. Here, we investigated the prognostic factors of EBV from 63 cases with relatively complete clinical profiles in Sierra Leone. Using different statistical models, we found that age, chest pain, coma, confusion and viral load were associated with the prognosis of EVD, in which viral load could be one of the most important factors for the survival of the disease.

## Introduction

In the year of 2014, Ebola virus disease (EVD) was quickly widespread and caused the whole world to pay attention [1,2]. By the end of 2014, more than eleven thousand cases were reported from West African countries such as Guinea, Sierra Leone, Liberia, Senegal, Nigeria, and Mali [1,2]. However, Ebola did not stop in West Africa only, it has gone globally as cases were diagnosed in the United States and Spain [1,2].

The Ebola outbreak had many clinical management challenges due to its high fatality rate (45–90%) and easy transmission [3,4]. Because of this, health care professionals are put at great risk when helping Ebola infected patients [5]. Such risks are greatly higher than regular daily practices. Although supportive care such as the use of antibiotics and administrating intravenous fluids is believed to be helpful, there is no clinically approved treatment to Ebola [6,7]. The survival rate of EVD can increase in places with advanced medical care because of constant maintenance of blood pressure, body fluids volume and hydration [8]. However, the most severely affected countries, Guinea, Liberia and Sierra Leone, have very weak health systems, lack human and infrastructural resources, and have only recently emerged from long periods of conflict and instability, which makes it extremely difficult to prevent and treat the disease.

Previous studies have provided some information regarding the prognosis of EVD [9,10]. Bah et al. reported that EVD patients who were 40 years of age or older had a higher risk of death compared with those under the age of 40 years using Poisson regression analysis [9]. Also, the viral load appeared to be higher in non-survivors compared to survivors by univariate analyses [9]. Schieffelin et al. showed that EVD patients under the age of 21 years had a lower case fatality rate than those over the age of 45 years, and patients presenting with fewer than 10^5^ EBOV copies/ml had a lower case fatality rate than those with 10^7^ EBOV copies/ml [10]. Also, weakness, dizziness, and diarrhea were the symptoms that were significantly associated with a fatal outcome [10]. In addition, Towner et al. presented that viral load could be correlated with disease outcome [11]. However, these studies were conducted using relatively small number of patients. Therefore, independent research is required to confirm these findings. Moreover, since it is extremely difficult to obtain completely detailed patient information during the outbreak [12,13], and it is biased and wasting of data to rule out patients with relatively uncompleted information, it would be necessary to apply COX's proportional hazard model to deal with this kind of data set. In this study, we investigated the prognostic factors of EVD using various statistical models.

## Materials and Methods

### Ethics statement

The institutional review board at Beijing 302 Hospital and the Sierra Leone Ethics and Scientific Review Committee approved this project. These committees waived the requirement to obtain informed consent during the West African Ebola outbreak.

### Patients

From Oct. 1st, 2014 to Jan. 18th, 2015, the Chinese Medical Team in the Jui Government Hospital, Sierra Leone admitted 661 patients and 269 of them were diagnosed EVD. Noticeably, Jui Government Hospital was positioned as a holding center from Oct 1st, 2014 to Dec 31st, 2014, and upgraded as an Ebola Treatment Center (ETC) on Jan 1^st^, 2015. The admission of patients was coordinated by the National Emergency Response Center (NERC). First of all, venous blood of the patients was immediately taken for sampling. They were then hospitalized and the samples were examined in the Chinese portable biosafety level 3 laboratories within the Jui Government Hospital. While waiting for the test results, patients were treated based on their clinical presentations. All the patients were given oral rehydration salts as a routine treatment, and the dose was dependent on the severity of dehydration. Intravenous administration of supplements was given under certain conditions. Patients with headache and/or muscle pain were given Acetaminophen or Ibuprofen. Patients with fever were given Cefixime or anti-infective Ciprofloxacin and anti-malaria Compound Naphthoquine Phosphate Tablets. Patients with upper abdominal pain or burning sensation were given antacid drugs such as Ranitidine or Omeprazole. Patients feeling fretful or insomnia were given Diazepam. A few patients were offered intravenous lactated Ringer’s solution. All the treatments were performed in compliance with ‘Clinical management of patients with viral haemorrhagic fever A pocket guide for the front-line health worker’ (http://www.who.int/csr/resources/publications/clinical-management-patients/en/) and ‘Manual for the care and management of patients in Ebola Care Units/ Community Care Centres Interim emergency guidance’ (http://www.who.int/csr/resources/publications/ebola/patient-care-CCUs/en/). Patients confirmed with Ebola infection were reported to NERC, and were transported to other treatment centers before December 31^st^, 2014, or stayed in the special treatment zone of Jui Government Hospital after the hospital was upgraded to ETC. Ebola-negative patients were arranged for corresponding treatments or discharge.

### Data collection

Sierra Leone is one the most severely affected countries. However, due to its weak health system and lack of human resources, it was extremely difficult to obtain complete patient data during the outbreak. Holding center faced even worse situation because EVD confirmed patients were transferred to different ETCs after the diagnosis. Of the 269 EVD cases, 201 subjects were diagnosed when Jui Government Hospital functioned as a holding center. Only 7 patients were obtained relatively complete information. Sixty eight patients were confirmed EVD between January 1^st^ and January 18th, 2015 after the hospital was upgraded to ETC, in which 56 cases provided relatively complete information, and the other 12 patients died soon after the admission and did not give enough information for the study. Patient data included demographic information such as gender, age and job; epidemiological history of attending traditional funerals; history of contacting with EVD patients; symptoms; clinical signs, etc. Patients were sent to the hospital ward after the healthcare workers evaluated the general situations and filled out the Ebola case investigation form or viral hemorrhagic fever case investigation form formulated by NERC. The EVD Patient Observation Sheet was completed daily after routine check, and all the sheets were transported either via closed-circuit surveillance system or by WiFi after being photographed. In order to keep the results accurate, all the data was recorded and compared separately in the Excel database by two healthcare professionals. Discharged patients were followed by calling randomly to patients themselves or their families or related treatment centers and the information of their conditions (whether they were cured/ discharged or died, and the date of cure or death) were being accessed.

### Virus detection

Viral load was examined upon the admission to the hospital. Detection of virus was performed by the Chinese-CDC portable biosafety level 3 laboratories based on previously published method [14]. These laboratories accepted specimens from the Jui Government Hospital as well as specimens transported by NERC from other ETC and holding centers. After recording the sign-in information in the biosafety level 3 laboratory, technicians inactivated the specimens by incubating in the water bath at 60 degree for 1 hour. Then 50 ul of inactivated specimen was used to isolate RNA by Viral RNA Isolation Kit (Life Technologies, Grand Island, NY, USA) and DNA by Automatic DNA Extractor (Life Technologies, Grand Island, NY, USA). The isolated RNA and DNA were tested in biosafety level 2 laboratory. Thermal cycling parameters of the real-time reverse transcription-polymerase chain reaction were 30 min at 42°C followed by 10 min at 95°C and a 40 cycles of amplification. The range of cycle threshold was between 18.34 and 35.81 and the range of viral load was between 10^3^ and 3.9x10^8^ copies/ml in our study population.

### Statistical analyses

Data were analyzed by PASW statistics 18.0 software. Pearson's chi-squared test was applied to compare dichotomous variables. The Yates’s correction was applied when the expected (theoretical) frequency was <5 and ≥ 1. The survival curve was estimated by Kaplan-Meier method. The Cox proportional hazard model was used for multi-factor analysis.

## Results

### The study population

In our study population, businessman (17 cases) and students (13 cases) were the two major professions. Of the 63 patients, 27 subjects were non-survivors and 36 subjects were survivors; 35 subjects had clear contact history, in which 11 cases were through funerals and 10 cases were through family members. As shown in Table 1, common symptoms included fever (80.95%), diarrhea (49.21%), vomiting (50.79%), fatigue (90.48%), anorexia (87.30%), abdominal pain (49.21%), chest pain (55.56%), muscle pain (74.60%), joint pain (77.78%), headache (65.08%), cough (39.68%), difficulty in breathing (41.27%), difficulty in swallowing (42.86%), sore throat (38.10%), jaundice (26.98%), redeye (28.57%), skin rash (6.35%), hiccups (19.05%), pain behind eye (12.70%), coma (7.94%), confusion (14.29%), and bleeding (9.52%).

**Table 1:** Correlation between the symptoms and the prognosis (chi square test).

Symptom	Survivors/ Non survivors	Total	Proportion	OR-95%CI	OR	P value (χ^2^)	P value* (corrected)
Fever	27/24	51	80.95%	0.646–11.006	2.667	0.165	
Vomiting	19/13	32	50.79%	0.306–2.256	0.831	0.716	
Diarrhea	15/16	31	49.21%	0.739–5.614	2.036	0.167	
Fatigue	32/25	57	90.48%	0.265–9.230	1.563	0.620	0.951
Anorexia	29/26	55	87.30%	0.723–54.480	6.276	0.063	0.140
Abdominal Pain	19/12	31	49.21%	0.263–1.950	0.716	0.513	
Chest Pain	16/19	35	55.56%	1.033–8.532	2.969	0.040	
Muscle Pain	26/21	47	74.60%	0.420–4.312	1.346	0.616	
Joint Pain	28/21	49	77.78%	0.301–3.321	1.000	1.000	
Headache	24/17	41	65.08%	0.299–2.415	0.850	0.760	
Cough	16/9	25	39.68%	0.222–1.760	0.625	0.372	
Difficulty in Breathing	15/11	26	41.27%	0.349–2.653	0.963	0.941	
Difficulty in Swallowing	15/12	27	42.86%	0.409–3.068	1.120	0.825	
Sore Throat	13/11	24	38.10%	0.436–3.392	1.216	0.708	
Jaundice	9/8	17	26.98%	0.413–3.866	1.263	0.682	
Redeye	9/9	18	28.57%	0.499–4.505	1.500	0.469	
Skin Rash	2/2	4	6.35%	0.179–10.323	1.360	0.765	1.000
Hiccups	8/4	12	19.05%	0.162–2.281	0.609	0.459	
Pain Behind Eye	5/3	8	12.70%	0.168–3.570	0.775	0.743	1.000
Coma	0/5	5	7.94%	—	—	0.007	0.026
Confusion	2/7	9	14.29%	1.125–31.472	5.950	0.022	0.055
Bleeding	2/4	6	9.52%	0.500–17.496	2.957	0.215	0.421
Sex (male)	17/12	29	46.0%	0.328–2.436	0.894	0.827	
Age (≥40)	7/10	17	27.0%	0.782–7.592	2.437	0.120	
Viral load (>10^6^copies/ml)	13/20	33	52.4%	1.688–15.140	5.055	0.003	

*Data were corrected for continuity. OR, odd ratio; CI, confidence interval.

### One-way ANOVA analysis of the survival of EVD patients

**Fig 1:**
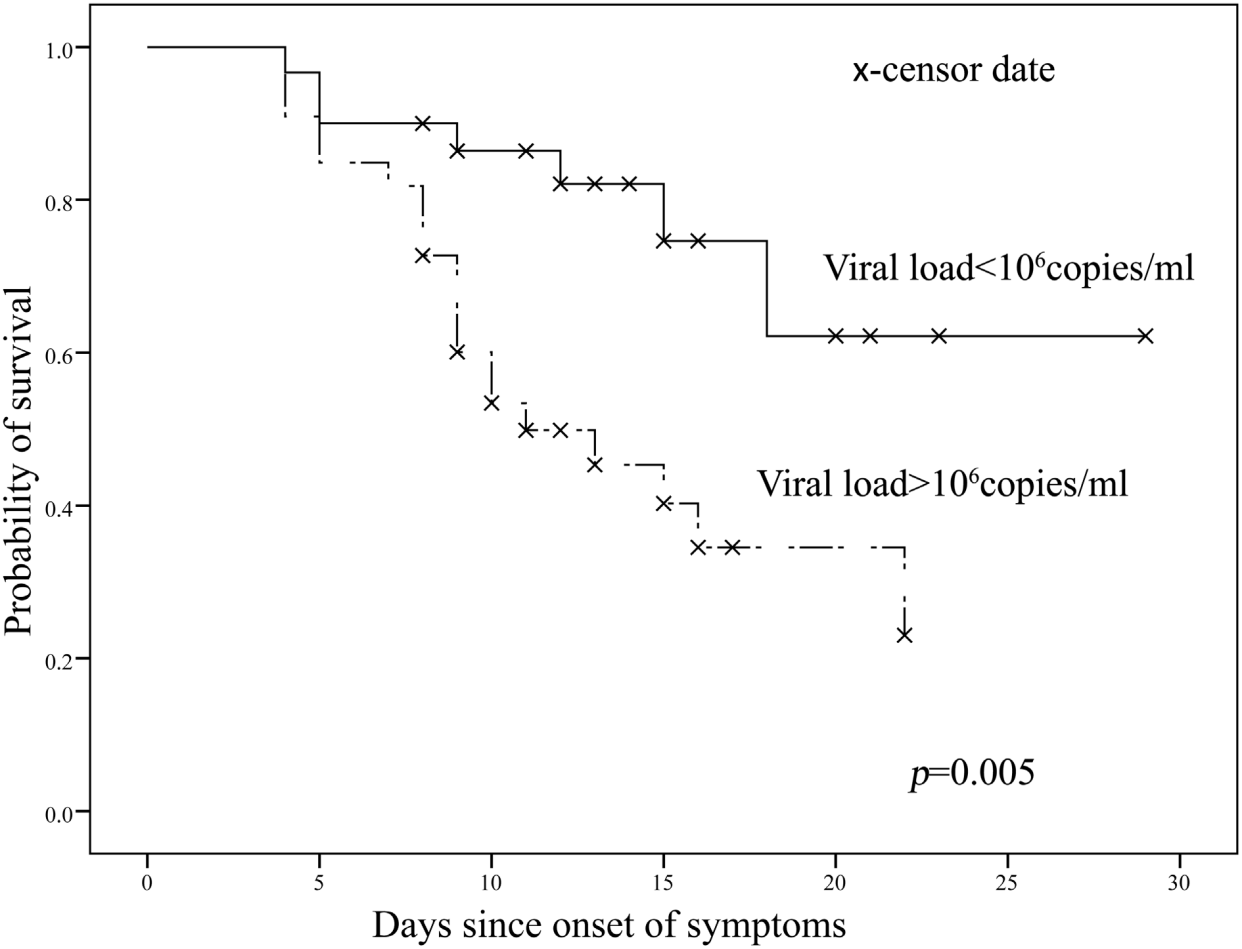
Kaplan-Meier survival analysis for EBV patients. Cases were divided into 2 group based on the viral load in their peripheral blood.

We first investigated patient’s age and the prognosis of EVD. Based on WHO and other literatures [9], patients were divided into two groups (≥40 years old and <40 years old). Log-rank test showed that the ≥40 years old group had moderately shorter survival time than the <40 years old group (P = 0.087). We further examined the viral load and the survival of EVD patients. Since 10^6^ is the closest integer to the median value of the viral load of our study population, we divided the cases into two subsets according to the value. Data showed that patients with viral titer higher than 10^6^ copies/ml presented significantly shorter survival time than those whose viral titer were lower than 10^6^ copies/ml (P = 0.005, Fig 1).

We also examined the correlation between clinical symptoms and the survival of EVD. Using Pearson chi-square test, we found that fever, diarrhea, vomiting, fatigue, anorexia, abdominal pain, chest pain, muscle pain, joint pain, headache, cough, difficulty in breathing, difficulty in swallowing, sore throat, jaundice, redeye, skin rash, hiccups, pain behind eye, coma, confusion, and bleeding were not significantly associated with EVD death, whereas symptoms such as chest pain, coma and confusion showed significantly association with EVD mortality (P = 0.040, P = 0.007, and P = 0.022, Table 1). However, value of confusion lost statistical significance after corrected for continuity (P = 0.055, Table 1). Furthermore, we analyzed the positive predictive values (PV+) and negative predictive values (PV-) of chest pain, coma and confusion. Data showed that chest pain presented 54.3% of PV+ and 71.4% of PV-; coma presented 100% of PV+ and 62.1% of PV-; confusion presented 60.6% of PV+ and 76.7% of PV-. In addition, we investigated whether coma, chest pain, confusion, and viral load were associated with each other using Pearson's chi-squared test. No significant difference was identified (coma vs. chest pain, p = 0.371; coma vs. confusion, p = 0.704; chest pain vs. confusion, p = 0.469, coma vs. viral load, p = 0.286; chest pain vs. viral load, p = 0.269; confusion vs. viral load, p = 0.847).

### Prognostic analysis by Cox proportional hazards model

One-way ANOVA analysis sometimes cannot reflect the combined effect of multiple variables on EVD. Since it is difficult to obtain complete patient information during the outbreak of EVD, whereas it is biased and wasting of data to rule out patients with relatively non-detailed information, applying COX's proportional hazard model to deal with this kind of data set is necessary. We found that that patient’s age, the symptom of confusion, and viral load were the significantly associated with the survival of EVD cases (P = 0.017, P = 0.002, and P = 0.027, respectively, Table 2).

**Table 2:** Survival analysis using Cox proportional hazards model.

Variables	β	SE	Wald	df	Sig.	Exp (β)
Age	0.030	0.013	5.701	1	0.017	1.031
Confusion	0.594	0.189	9.886	1	0.002	1.812
Log_10_ (Viral load)	1.086	0.491	4.899	1	0.027	2.964

β, beta coefficient; SE, standard error; Wald, Wald statistic; df, degree of freedom; Sig., P value.

## Discussion

In this study, we identified significant differences between survivors and non-survivors in terms of chest pain and coma. Moreover, the p value was close to 0.05 for symptoms such as diarrhea, anorexia and fever. These data indicate that the current Ebola outbreak is similar to previous ones [15,16]. Meanwhile, we observed some differences. First of all, EVD used to be called Ebola hemorrhagic fever, but most cases did not show bleeding or just had little fever in the current Ebola outbreak. It might be due to different strains of the Ebola virus. Secondly, occurrence of chest pain, coma and confusion were statistically significantly correlated with EVD death by Pearson’s chi-squared test (Table 1), whereas only confusion showed correlation to the survival of EVD by Cox proportional hazard model (Table 2). There could be two reasons causing the discrepancy: 1) sample size was too small; 2) Cox proportional hazard model takes survival time into consideration. Different survival time had different effects on the model, even though the clinical outcome of the patients was the same, whereas one-way ANOVA analysis only considers the clinical outcome of the patients.

In terms of age and the prognosis, we found that the survival time was shorter and the mortality was higher in older people through various statistical analyses. These data suggest that we should pay more attention to elderly patients and give them more efficient treatments. In terms of viral load and prognosis, we found that shorter survival time and higher mortality happened to the patients with higher viral load, when the patients were at similar age. These results suggest that it might be important to increase the efficiency of anti-viral treatment in order to lower the mortality and improve prognosis. Although the existing anti-viral drug such as Zmapp seems to be effective, we need larger scale clinical trials to prove it.

We treated the patients mainly with oral therapy. Although we evaluated the severity of dehydration by asking and observing the amount of urine, the amount of vomiting, heart rate, the condition of peripheral limbs circulation and skin elasticity, it was difficult to adjust the dosing of the drugs without biochemical test and blood routine test. This also happened in other treatment centers [17,18]. There were several reasons causing the lack of intravenous therapy. First of all, we had a shortage of experienced healthcare personnel. Similar to previous disease outbreak, many healthcare staff, especially the well-trained and skilled nurses, got infected at the beginning of the outbreak, and were forced to leave their positions [19]. Secondly, different from routine medical work, it was mandatory for the healthcare staff to wear multi-layered personal protective equipment (PPE) when treating the patients. The multi-layered gloves and goggles made the intravenous injection much more difficult.

There are some difficulties in conducting the research. At the beginning of the Ebola outbreak, the Jui Government Hospital was in paralysis as lots of experienced professionals left their positions. The data were messy as there was no formulated mode to record patients’ information. It turned better after we trained the medical team repeatedly. However, it was difficult to statistically compare the mortality with that from other reports since the outcome of the patients remained largely unknown. Also, as some of the local people don’t speak English, the communication with the Chinese doctors was through the translation from Sierra Leone nurses, which increased the possibility of misunderstanding and led to misjudgment of some symptoms. In addition, before upgraded to ETC, Jui Government Hospital transported most of the patients confirmed with Ebola virus to other ETCs, which made it difficult to get access to the treatment and outcome of these patients.

In conclusion, our data indicate that clinicians should pay close attention and give efficient treatment for elderly EVD patients and whose with high viral load. Future studies should focus on how to carry out intravenous therapy efficiently and safely as well as to develop novel antiviral drugs.

## Supporting Information

S1 Checklist STROBE checklist. (DOC)
